# Delafloxacin-Capped Gold Nanoparticles (DFX-AuNPs): An Effective Antibacterial Nano-Formulation of Fluoroquinolone Antibiotic

**DOI:** 10.3390/ma15165709

**Published:** 2022-08-18

**Authors:** Amr Selim Abu Lila, Bader Huwaimel, Ahmed Alobaida, Talib Hussain, Zeeshan Rafi, Khalid Mehmood, Marwa H. Abdallah, Turki Al Hagbani, Syed Mohd Danish Rizvi, Afrasim Moin, Abobakr F. Ahmed

**Affiliations:** 1 Department of Pharmaceutics, College of Pharmacy, University of Ha’il, Ha’il 81442, Saudi Arabia; 2 Department of Pharmaceutics and Industrial Pharmacy, Faculty of Pharmacy, Zagazig University, Zagazig 44519, Egypt; 3 Department of Pharmaceutical Chemistry, College of Pharmacy, University of Ha’il, Ha’il 81442, Saudi Arabia; 4 Department of Pharmacology and Toxicology, College of Pharmacy, University of Ha’il, Ha’il 81442, Saudi Arabia; 5 Nanomedicine and Nanotechnology Lab, Department of Biosciences, Integral University, Lucknow 226026, India; 6 Department of Pharmacy, Abbottabad University of Science and Technology, Havelian 22500, Pakistan; 7 Department of Microbiology and Immunology, Faculty of Pharmacy, Minia University, Minia 61519, Egypt

**Keywords:** antibiotic resistance, delafloxacin, fluoroquinolone, gold nanoparticles, nano-formulations

## Abstract

New antibiotics are seen as ‘drugs of last resort’ against virulent bacteria. However, development of resistance towards new antibiotics with time is a universal fact. Delafloxacin (DFX) is a new fluoroquinolone antibiotic that differs from existing fluoroquinolones by the lack of a protonatable substituent, which gives the molecule a weakly acidic nature, affording it higher antibacterial activity under an acidic environment. Furthermore, antibiotic-functionalized metallic nanoparticles have been recently emerged as a feasible platform for conquering bacterial resistance. In the present study, therefore, we aimed at preparing DFX-gold nano-formulations to increase the antibacterial potential of DFX. To synthesize DFX-capped gold nanoparticles (DFX-AuNPs), DFX was used as a reducing and stabilizing/encapsulating agent. Various analytical techniques such as UV-visible spectroscopy, TEM, DLS, FTIR and zeta potential analysis were applied to determine the properties of the synthesized DFX-AuNPs. The synthesized DFX-AuNPs revealed a distinct surface plasmon resonance (SPR) band at 530 nm and an average size of 16 nm as manifested by TEM analysis. In addition, Zeta potential results (−19 mV) confirmed the stability of the synthesized DFX-AuNPs. Furthermore, FTIR analysis demonstrated that DFX was adsorbed onto the surface of AuNPs via strong interaction between AuNPs and DFX. Most importantly, comparative antibacterial analysis of DFX alone and DFX-AuNPs against Gram-negative (*Escherichia coli* and *Pseudomonas aeruginosa*) and Gram-positive (*Staphylococcus aureus* and *Bacillus subtilis*) verified the superior antibacterial activity of DFX-AuNPs against the tested microorganisms. To sum up, DFX gold nano-formulations can offer a promising possible solution, even at a lower antibiotic dose, to combat pathogenic bacteria.

## 1. Introduction

Resistance to quinolones is a common issue since the early introduction of nalidixic acid into clinical practice in 1962. In the early 90s, the use of fluoroquinolones in the United States has climbed by ~40%, with doubling in the resistance rate to various fluoroquinolones [1,2]. Nowadays, sharp upsurges in fluoroquinolones resistance have been observed due to modification in target enzymes (DNA gyrase and topoisomerase IV), and development of efflux mechanism in pathogens [2,3]. Delafloxacin (DFX) is a novel broad-spectrum fluoroquinolone antibiotic that has been approved by the FDA in the year 2017. DFX has demonstrated strong bactericidal activity against both aerobic Gram-positive and Gram-negative pathogens [3,4,5]. DFX was also considered to be effective against anaerobes (Bacteroides fragilis and Clostridium perfringens) and a few typical organisms (Chlamydophila pneumonia and Legionella pneumonia) [3,6]. Like other fluoroquinolone antibiotics, DFX disrupts the topology and replication process of bacterial DNA by inhibiting DNA gyrase and topoisomerase IV activity [3]. Whilst, unlike other fluoroquinolone antibiotics, DFX have improved efficacy and absorption than other fluoroquinolones due to its acidic nature and being unionized in an acidic environment [7]. Nevertheless, it is a universal fact that pathogens could develop resistance towards a newer generation of antibiotics with time, and there is a huge plausibility that pathogens will find a way to become DFX-resistant in near future.

Nanotechnology makes it possible to study and control a wide range of biological and pharmacological processes that happen at the nanoscale level [8,9,10]. By cautiously altering the nano-structures, their solubility, biocompatibility, stability and molecule binding ability could be enhanced. The development of hybrid systems made up of nanoparticles and biological molecules opens the door to a wide range of applications, such as biosensing, bioimaging and targeted drug delivery [11,12,13,14,15,16]. In addition, hybrid systems have been proved to improve oral delivery by increasing the bioavailability of the drugs that are not well absorbed [17]. In fact, nanoparticles can permeate the connective tissue, which allows the drug/antibiotic to get to the right place without blocking the capillaries [18].

Recently, numerous nanoparticles with biomedical applications have been explored extensively, such as metallic nanoparticles, polymeric nanoparticles, micelles, quantum dots, liposomes and dendrimers [19,20,21,22,23,24,25]. Among them, metallic nanoparticles, particularly gold nanoparticles (AuNPs), have emerged as appealing delivery vehicles for antibacterial drugs [26,27,28]. Upon loading/capping of antibacterial agents onto gold nanoparticles (AuNPs), antibacterial agents have been reported to exert better antibacterial activity, which might aid in the fight against resistant bacteria. For example, the antibacterial activity of ampicillin was restored against several resistant strains of *Enterobacter aerogenes* and *Pseudomonas aeruginosa* after being loaded onto gold nanoparticles [29]. In the same context, Al Hagbani et al. [9] have recently demonstrated that capping of cefotaxime onto AuNPs significantly augmented the antimicrobial activity of capped cefotaxime against Gram-positive (*Staphylococcus aureus*) and Gram-negative (*Escherichia coli*, *Klebsiella oxytoca*, *Pseudomonas aeruginosa*) bacteria, as evidenced by significantly lower minimum inhibitory concentration (MIC) values compared to plain cefotaxime. Collectively, AuNPs-based antibiotic delivery systems might provide a plausible solution to combat pathogenic bacteria and/or circumvent antimicrobial resistance.

**Figure 1:**
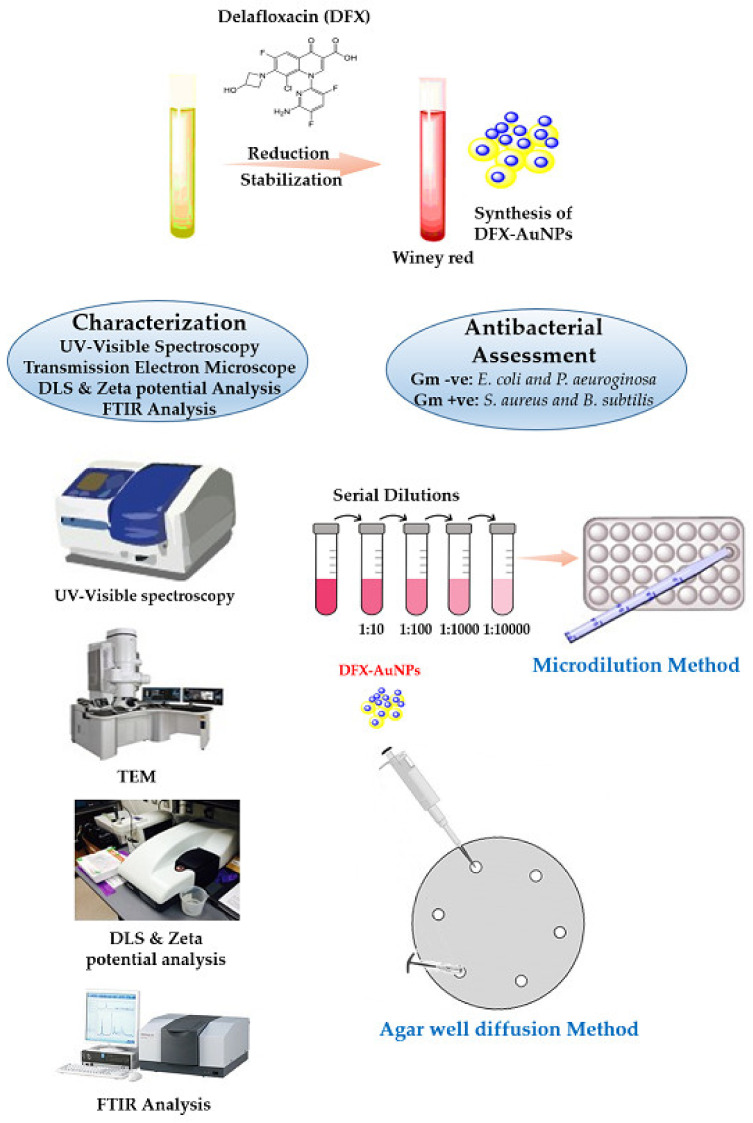
Schematic representation of DFX-AuNPs synthesis procedure, its characterization and antibacterial potential analysis.

The aim of this study, therefore, was to prepare delafloxacin-gold nano-formulation to increase the antibacterial potential of DFX. A one-pot synthesis approach for the fabrication of DFX-AuNPs was adopted wherein DFX was exploited as a reducing and capping agent to convert gold salts into AuNPs, rather than using an external reducing/capping agent. In addition, a variety of analytical techniques including UV-visible spectrum, transmission electron microscopic analysis, dynamic light scattering, and zeta-potential analysis were applied to characterize DFX-AuNPs. Further, the amount of DFX loaded on the AuNPs was estimated prior to antibacterial assessment against different bacterial strains. The schematic representation of work performed in the current study has been shown in Figure 1.

## 2. Materials and Methods

### 2.1. Materials

All of the solvents, antibiotics, and components utilized in the current investigation were of analytical quality and procured from Sigma Aldrich (St. Louis, MO, USA). Mueller–Hinton (MH) agar was purchased from Hi-media (Mumbai, India).

### 2.2. Synthesis of DFX-AuNPs

AuNPs were prepared by incubation of 3 mL reaction mixture containing 1 mM gold salt (H[AuCl_4_]) solution [in phosphate buffer (pH = 7.4)] and 333.3 µg/mL DFX. After that, further incubation of the samples was done at 40 °C for 48 h. Reaction batch containing solely DFX was used as a reference. After 48 h of incubation, the solution’s color changed to ruby red, suggesting that the product (AuNPs) has been formed.

### 2.3. Confirmation of Formation of DFX-AuNPs

The reduction process of gold salt into AuNPs was followed up by using dual-beam UV spectrophotometer (Shimadzu UV-1601, Tokyo, Japan). It relied on the fact that transformation of gold salts into AuNPs will show a characteristic surface plasmon resonance (SPR) band. The synthesized DFX-AuNPs were further evaluated on the basis of their hydrodynamic diameter by utilizing the Dynamic Light Scattering (DLS) technique on a Zeta sizer Nano-ZS (ZEN3600, Malvern Instrument Ltd., Malvern, UK). The zeta potential (particle surface charge) of DFX-AuNPs was measured using Malvern Zeta sizer Nano-ZS. Transmission electron microscopy (TEM; Tecnai G2 Spirit) equipped with a Bio Twin lens configuration and 80 kV of accelerating voltage was used to estimate the size and shape of synthesized DFX-AuNPs.

### 2.4. Comparative FTIR Analysis of DFX and DFX-AuNPs

The Jasco FTIR spectrophotometer (Tokyo, Japan) was employed to record the FTIR spectra of pure DFX and DFX-AuNPs. For the analysis, each test sample was powdered with potassium bromide (10:100) and then compressed to form a transparent film. The spectra were logged in the wavelength range of 4000 to 400 cm^−1^ and respective peaks were construed and analyzed with the help of Spectra Manager™ 2.5 software.

### 2.5. Loading Efficiency Determination

A 30-min centrifugation at 30,000× *g* was used to extract the produced DFX-AuNPs from the reaction mixture, and the supernatant was used to evaluate DFX loading efficiency onto DFX-AuNPs. The total amount of free DFX in the supernatant was quantified spectrophotometrically at a maximum wavelength of 290 nm. The following equation was applied to calculate the loading efficiency:

$$\mathrm{Loading}\;\mathrm{Efficiency}\;\left(\%\right)=\frac{{\mathrm{DFX}}_{\mathrm{total}}-{\;\mathrm{DFX}}_{\mathrm{free}}}{{\mathrm{DFX}}_{\mathrm{total}}}\times 100$$

where, DFX_total_ represents the entire quantity of DFX added throughout the synthesis of DFX-AuNPs, and DFX_free_ represents the quantity of free DFX that is present in the supernatant of DFX-AuNPs.

### 2.6. Antibacterial Assessment

#### 2.6.1. Bacterial Culture and Growth Conditions

Gram-negative (*Escherichia coli* ATCC 25922, *Pseudomonas aeruginosa* NCIM 2036) and Gram-positive (*Staphylococcus aureus* ATCC 25923, *Bacillus subtilis* MTCC 441) bacterial strains were used to determine antibacterial efficacy of DFX-AuNPs. Each bacterial strain was transferred to a fresh inoculum in Luria–Bertani broth (LB), and further incubation was done for 20 h at 37 °C. Prior to antibacterial analysis, the culture was diluted to a turbidity of 0.5 McFarland standard, which corresponds to 1.5 × 10^8^ CFU/mL, using LB broth.

#### 2.6.2. Preliminary Antibacterial Analysis via Agar Well Diffusion Method

Antibacterial activity was assessed by the agar well-diffusion method, which involved addition of microbial inoculum (100 µL) uniformly over the agar plates. The entire surface of the agar plates was then swabbed three times, and petri plates were rotated at 60° angle after each application. Subsequently, 100 µL (29.33 µg/well) of each sample (DFX and DFX-AuNPs) was poured into the 6 mm wells punched into agar plates via well cutter. Phosphate buffer saline (pH 7.4) was employed as a control. The plates were analysed to examine the zone of inhibition in millimeters (mm) after overnight incubation at 37 °C. Three replica tests were conducted for pure DFX and DFX-AuNPs to minimize errors associated with comparative antibacterial activity results.

#### 2.6.3. Minimal Inhibitory Concentration (MIC) Estimation by Micro-Dilution Method

The broth micro-dilution method [9] was employed to assess the MIC of pure DFX and synthesized DFX-AuNPs against the tested bacterial strains. To summarize, 10 µL of each bacterial inoculum (1 × 10^5^ CFU/mL) were added to each well in a 96-well plate. Subsequently, serial dilutions in the concentration range of 3.90–250 µg/mL of the synthesized DFX-AuNPs were added to each well. At the same concentration, pure DFX is also added for comparative analysis on a separate 96-well plate. Both DFX- and DFX-AuNPs-treated plates were incubated overnight at 37 °C, and cell viability was determined at 625 nm using ELISA plate reader. The MIC was recorded as the minimum quantity of DFX-AuNPs or free DFX that effectively inhibited bacterial growth after overnight incubation. During the experiment, phosphate buffer saline (pH 7.4) was used as a negative control. The obtained results were presented as mean ± SD of three independent triplicates.

## 3. Results and Discussion

### 3.1. UV-Visible Spectroscopy Analysis

**Figure 2:**
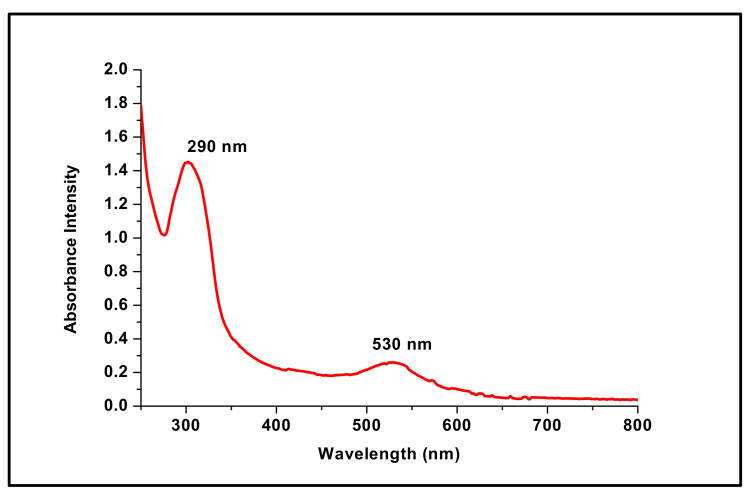
UV-Visible spectroscopy of DFX-AuNPs showing SPR band at 530 nm.

Gold nanoparticles have a unique optical property known as localized surface plasmon resonance, resulting in a characteristic absorbance band in the visible region (500–600 nm), which can be measured by UV-Vis spectroscopy. In this study, a characteristic absorbance peak at 530 nm was observed in the UV-Vis spectrum of the reaction mixture (Figure 2). Importantly, it was within the characteristic plasmon peak range of AuNPs, indicating the successful synthesis of AuNPs [30]. In addition, the presence of an additional peak at 290 nm (Figure 2), which is specific to the DFX molecules [31], confirmed the efficient loading of DFX onto the surface of AuNPs. Notably, the color change of the reaction mixture from light yellow to the characteristic wine-red color also confirmed the formation of DFX-loaded AuNPs. Collectively, these findings clearly confirm the efficient attachment/capping of DFX molecules on the AuNPs surface and underscore the applicability of DFX as an effective reducing agent, resulting in the reduction of gold salt to AuNPs in a controlled manner.

### 3.2. Characterization of DFX-AuNPs

The characterization of nanosystems traditionally relies on the determination of morphology, size and surface charge using various analytical techniques

#### 3.2.1. Electron Microscopy and Dynamic Light Scattering Analysis of DFX-AuNPs

**Figure 3:**
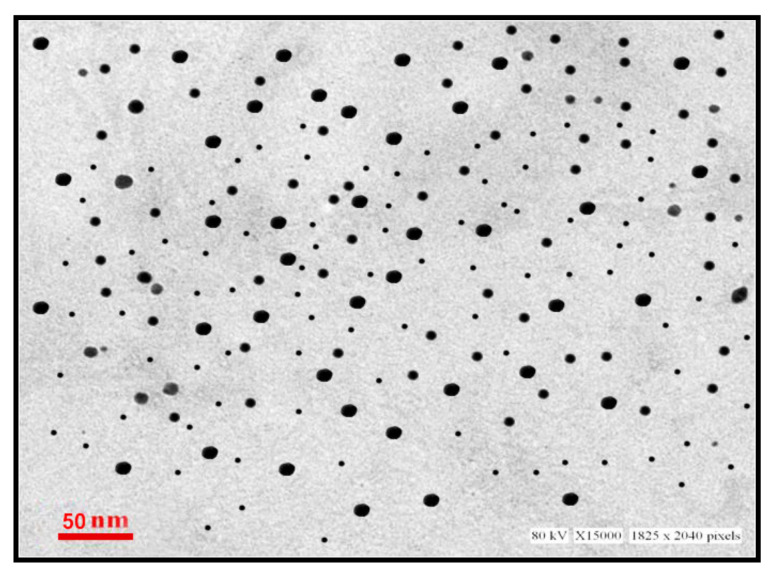
TEM micrograph of DFX-AuNPs.

Transmission electron microscope (TEM) is a powerful tool for attaining information such as morphology, particle size, and size distribution of nanoparticles. As shown in Figure 3, DFX-AuNPs were spherical in morphology and are monodispersed with an average size of 16 nm. Notably, no agglomeration/aggregation was found in the TEM micrographs, suggesting the effectiveness of delafloxacin as a capping/stabilizing agent.

#### 3.2.2. Dynamic Light Scattering and Zeta-Potential Analysis

**Figure 4:**
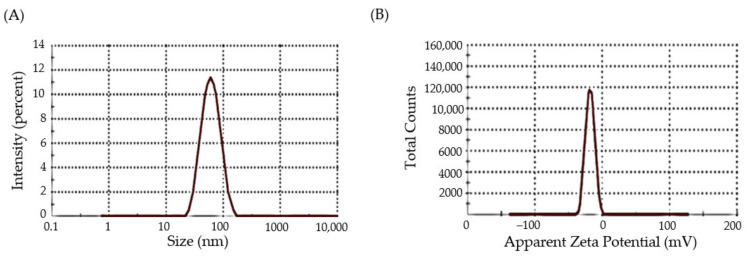
(**A**) DLS profile of DFX-AuNPs (80 nm) (**B**) Zeta-potential of DFX-AuNPs (−18 mV).

Dynamic light scattering (DLS) is a well-established technique for determining the size and size distribution of nanoparticles in a dispersion [32]. The random change in the intensity of light scattered from a dispersion of nanoparticles is utilized to calculate particle size. As depicted in Figure 4A, DFX-AuNPs showed an average hydrodynamic size as 80 nm. The comparatively larger size of DFX-AuNPs assessed by DLS (80 nm) with regard to that assessed by TEM (16 nm) might be accounted to the use of dispersant in DLS. In general, particle size estimate in TEM is done in the dry condition; TEM estimates the precise size of individual particles while ignoring the influence of the attached dispersant’s solvent layer. By contrary, the DLS technique calculates particle size as a hydrodynamic diameter (hydrated state). As a result, the particles will have a greater hydrodynamic volume owing to the solvent effect in the hydrated state.

Zeta potential estimation is another important characterization technique for the assessment of surface charge of nanoparticles [33]. It represents a key indicator for the stability of colloidal dispersions; where the magnitude of the zeta potential reflects the degree of electrostatic repulsion between similarly charged adjacent particles in a dispersion. As shown in Figure 4B, DFX-AuNPs had a zeta potential value of −19 mV, depicting a significant stability of DFX-AuNPs. These results, along with the efficient capping effect of DFX, suggest the higher stability of DFX-AuNPs, as manifested by the absence of aggregation/agglomeration as observed in TEM images.

#### 3.2.3. Fourier Transform Infrared (FTIR) of Pure DFX and DFX-AuNPs

**Figure 5:**
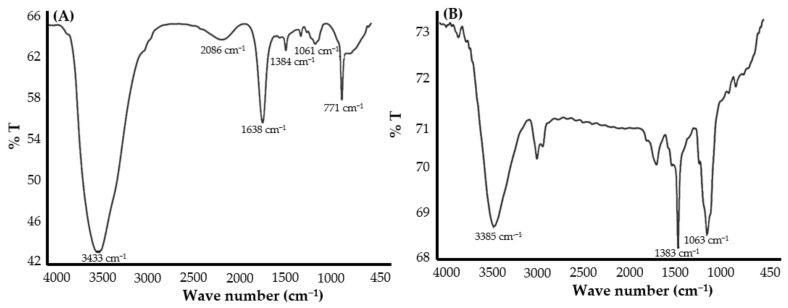
FTIR spectra of (**A**) pure DFX and (**B**) DFX-AuNPs.

FTIR studies can be adopted to recognize the possible agents/molecules responsible for capping and efficient stabilization of the metal nanoparticles. In this study, FTIR spectral analysis was obtained for the confirmation of attachment of DFX molecules over the surface of AuNPs. The major peaks recorded in the pure DFX sample were attributed to the functional groups: –O–H stretching (3433 cm^−1^), carboxylic –C=O stretching (1638 cm^−1^), C–F stretching (1061 cm^−1^) and C–Cl stretching (771 cm^−1^), which confirms the purity of the DFX antibiotic [34]. However, post synthesis of DFX-AuNPs, an elevation in the –O–H stretching peak intensity along with reduction in peak intensities at the respective –C=O stretching, C–F stretching and C–Cl stretching positions were observed (Figure 5A,B), which was attributed to the conformational change occurred in the DFX after capping onto AuNPs. Moreover, nitrogen of piperazinyl group of DFX might have played an important role in the interaction with AuNPs. Similar to the present study, other authors [35,36] also suggested that fluoroquinoloid antibiotics (norfloxacin, ciprofloxacin, and gatifloxacin) might interact with AuNPs via nitrogen of the piperazinyl group.

#### 3.2.4. Drug Loading Efficiency

The loading efficiency of nanoparticles is considered as a critical attribute for their characterization. The loading efficiency of DFX onto AuNPs was quantified as the proportion of DFX that was successfully loaded/attached to the nanoparticles surface. Our results depicted a significant loading efficiency of ~88%. Initially, 333.3 µg/mL of DFX was used in the process, and 293.3 µg/mL of the DFX had been significantly attached onto the surface of AuNPs.

### 3.3. Antibacterial Efficacy of DFX-AuNPs

After successful synthesis and characterization of DFX-AuNPs, antibacterial investigations were carried out against different Gram-positive and Gram-negative bacterial strains. Pure DFX was used for comparison, to depict the change in its efficacy after loading onto AuNPs. Table 1 shows the growth inhibition (agar well-diffusion method) induced by DFX-AuNPs and pure DFX against bacterial strains of *Escherichia coli*, *Pseudomonas aeruginosa*, *Staphylococcus aureus* and *Bacillus subtilis*. When compared with pure antibiotic DFX, the antibacterial capabilities of DFX-AuNPs were observed to be significantly higher. The obtained zone of inhibition (in mm) is represented in Table 1.

### 3.4. Determination of Minimal Inhibitory Concentration of CTX and C-AuNPs

**Figure 6:**
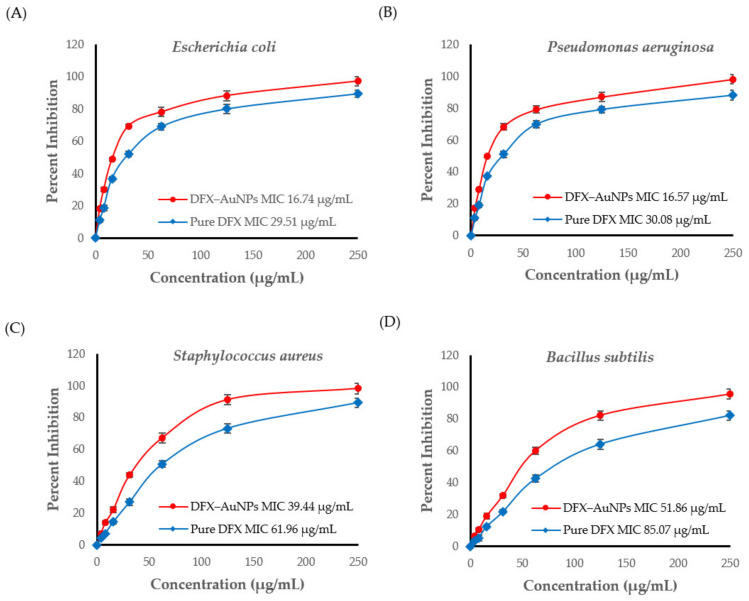
Minimal inhibitory concentration (MIC) curves of DFX and DFX-AuNPs obtained against (**A**) *Escherichia coli*; (**B**) *Pseudomonas aeruginosa*; (**C**) *Staphylococcus aureus* and (**D**) *Bacillus subtilis*. Data represent mean ± standard deviation of three independent experiments conducted under identical experimental conditions.

The results obtained from agar well-diffusion prompted to estimate the MIC (Figure 6) for DFX and DFX-AuNPs against the tested bacterial strains. The MICs of pure DFX were found to be 29.51 µg/mL, 30.08 µg/mL, 61.96 µg/mL and 85.07 µg/mL against *Escherichia coli*, *Pseudomonas aeruginosa*, *Staphylococcus aureus* and *Bacillus subtilis*, respectively. However, the MICs of DFX-AuNPs were 16.74 µg/mL, 16.57 µg/mL, 39.44 µg/mL and 51.86 µg/mL against *Escherichia coli*, *Pseudomonas aeruginosa*, *Staphylococcus aureus* and *Bacillus subtilis*, respectively.

Antibacterial activity results (Table 1 and Figure 6) showed that DFX has become more potent after loading onto AuNPs. MIC results (Figure 6) showed that DFX-AuNPs were more efficient against Gram-negative organisms. Similarly, Shamaila et al. [37] also observed enhanced activity of chemically synthesized gold nanoparticles against Gram-negative enteric pathogens than Gram-positive pathogens. This might be due the structural differences in Gram-negative and Gram-positive pathogens. It has been reported by several authors that AuNPs could act as an effective delivery tool for antibiotics with enhanced bactericidal potential irrespective of their Gram typing [9,28,38,39]. Owing to their tiny size and great penetrating capability, AuNPs could successfully bind to the substrates on the outer and cell membranes of the pathogens [38,40]. In addition, AuNPs could form irreversible pores in the membrane [40]. Another advantage of AuNPs as a carrier is that it provides enormous surface area to transport a large quantity of antibiotic moiety. Thus, it could be speculated that in the present study, AuNPs delivered more quantity of DFX to the tested bacterial strains as compared to DFX alone.

During the last few decades, quinolones have evolved from a minor and unimportant family of drugs used exclusively to treat urinary tract infections to some of the most routinely prescribed antibiotics in the world [41,42,43]. They are currently used to treat a broad spectrum of both Gram-positive and Gram-negative bacterial infections. Nevertheless, the use of quinolones is unfortunately endangered by the increasing development of resistance, which has been observed in many clinical settings. The fluoroquinolones resistance mechanism involves the target modification (DNA gyrase in Gram-negative or topoisomerase IV in Gram-positive pathogens) and efflux mechanism [2]. Loading antimicrobial agents onto AuNPs was reported to evade bacterial resistance mechanisms. In a recent report, AuNPs acted synergistically with ciprofloxacin to inhibit efflux pump in multi-drug-resistant Gram-negative pathogens [44]. In the present study, therefore, we tested the feasibility of nanotechnology to augment the antibacterial efficacy of the fluoroquinolone, delafloxacin, via its loading onto gold nanoparticles (AuNPs) and testing its antibacterial potential against different Gram-positive and Gram-negative bacteria. A one-pot synthesis approach for the fabrication of DFX-AuNPs was adopted, wherein DFX was exploited as a reducing and capping agent to convert gold salts into AuNPs, rather than using an external reducing/capping agent. Furthermore, comparative antibacterial analysis of DFX alone and DFX-AuNPs against Gram-negative and Gram-positive bacteria verified the superior antibacterial activity of DFX-AuNPs against the tested microorganisms. Collectively, it can be safely proposed that AuNPs as an efficient drug carrier for DFX might be used to decrease/overcome the risk of DFX resistance in near future.

## 4. Conclusions

The present study established a facile one-pot method for the synthesis of delafloxacin-loaded gold nanoparticles (DFX-AuNPs) via exploiting the reducing and capping power of DFX. The produced DFX-AuNPs was discrete, spherical in shape, and was in the nano-size range. The drug-loading efficiency was ~88%. Most notably, the antibacterial investigation revealed that loading DFX onto the surface of AuNPs remarkably increased its antibacterial efficacy against tested Gram-negative and Gram-positive bacterial strains, even at lower doses than pure DFX. To sum up, the current study paves the path for the simultaneous production of many AuNPs-based antibiotic formulations in an attempt to eliminate the issue of rising resistance. Nevertheless, further in vivo investigations to assess the fate and toxicity of DFX-AuNPs are urgently needed before reaching a compelling conclusion on the applicability of synthesized delafloxacin nano-formulations.

## Figures and Tables

**Table 1:** Zone of inhibition obtained through agar well diffusion method.

Zone of Inhibition (mm)				
	Gram-Negative		Gram-Positive	
Sample	*E. coli*	*Ps. aeruginosa*	*S. aureus*	*B. subtilis*
Pure DFX	20.1 ± 1.2	18.2 ± 0.9	17.3 ± 0.8	19.2 ± 1.3
DFX-AuNPs	32.3 ± 1.5	29.1 ± 1.0	31.4 ± 0.9	33.1 ± 0.6

Data represent mean ± standard deviation of three independent experiments conducted under identical experimental conditions.

## Data Availability

Not applicable.
